# Handbook for the Physiological Laboratory

**Published:** 1873-07

**Authors:** 


### Books Reviewed.

*Handbook for the Physiological Laboratory.* By E. Klein, M. D., J. Burdon-Sanderson, M. D., F. R. S.; Michael Foster, M. A., M. D., F. R. S., and T. Lauder Brunton, M. D., D. Sc. Edited by J. Burdon-Sanderson. Two volumes with 353 illustrations. Vol, I., Text. Vol. II., Illustrations. Philadelphia: Lindsay & Blakiston, 1873.

Of the many books which have been lately brought to the notice of the profession we know of none which is more admirably calculated to fill the place designed for it than the one under consideration. The editor informs us that it is intended as a hand-book for beginners in physiological investigation. For this purpose it is better calculated to guide and instruct the student than is any single work with which we are acquainted. Written by men of acknowledged ability, in their several departments, it may not only serve as a guide to the student but will undoubtedly find a prominent position in the list of text books to which the more advanced and experienced may desire to refer.

The first part is from the pen of Dr. Klein, a writer whose knowledge and experience in Histology is undisputed. This section is devoted to histology, the subjects which are considered being: The blood, epithelium and endothelium, connective tissues, muscular tissues, tissues of the nervous system, the methods of preparation of compound tissues; the vascular system, the lymphatic system, organs of respiration and digestion, the skin, cutaneous glands, and genito-urinary apparatus; organs of special sense, embryology and in the form of an appendix a study of inflamed tissues. Embracing as this does the whole subject and being written in so plain and concise terms the reader can, with but little difficulty, follow the author in his laboratory practice and be enabled to achieve results which, without the plain, practical guidance of Dr. Klein, would be impossible to him.

Part Second, on Physiology, will not in the least detract from the interest in the work which section first may have excited. The first part of this section is by Dr. Burdon-Sanderson, and is devoted to a consideration of the blood, circulation, respiration and animal heat. The blood is treated of under the following heads: Liquor, sanguinis, conditions effecting coagulation of blood, coloring matter, and gasses of the blood; all of which are presented in plain and simple terms. The various methods of analysis which will give the most exact results are also dwelt upon in the concluding paragraphs of this chapter. The circulation of the blood, together with the instruments for making observations concerning it, is described in the sixteenth chapter. Respiration and animal heat are fully described in the following two chapters.

Part second of the section on Physiology, by Dr. Michael Foster, is a consideration of the functions of muscle and nerve. The different subjects embraced are each treated to sufficient length and in sufficiently clear terms; ample reference is made to the volume of illustrations, and the methods of experimental research are in precise and well selected language.

Dr. Lauder Brunton is the author of the remaining chapters on Physiology which treats of digestion and secretion. A larger number of experimens are given, particular attention being drawn to the more valuable and easily tried by an asterisk or dagger. Following this is an appendix giving practical notes on manipulation, which will add in a large measure to the value of the work. The volume of illustrations are well suited to explain the text, and although we do not like the idea of presenting them separate from the reading matter their general excellence forbids any fault being found.

